# Rocking at 81 and Rolling at 34: ROC Cut-Off Scores for the Negative Acts Questionnaire–Revised in Serbia

**DOI:** 10.3389/fpsyg.2016.02058

**Published:** 2017-01-09

**Authors:** Ivana B. Petrović, Milica Vukelić, Svetlana Čizmić

**Affiliations:** Department of Psychology, Faculty of Philosophy, University of BelgradeBelgrade, Serbia

**Keywords:** workplace bullying, Negative Acts Questionnaire–Revised (NAQ-R), receiver operating characteristic (ROC), cut-off points, Serbia

## Abstract

Researchers are still searching for the ways to identify different categories of employees according to their exposure to negative acts and psychological experience of workplace bullying. We followed Notelaers and Einarsen’s application of the ROC analysis to determine the NAQ-R cut-off scores applying a “lower” and “higher” threshold. The main goal of this research was to develop and test different gold standards of personal and organizational relevance in determining the NAQ-R cut-off scores in a specific cultural and economic context of Serbia. Apart from combining self-labeling as a victim with self-perceived health, the objectives were to test the gold standards developed as a combination of self-labeling with life satisfaction, self-labeling with intention to leave and a complex gold standard based on self-labeling, self-perceived health, life satisfaction and intention to leave taken together. The ROC analysis on Serbian workforce data supports applying of different gold standards. For identifying employees in a preliminary stage of bullying, the most applicable was the gold standard based on self-labeling and intention to leave (score 34 and higher). The most accurate identification of victims could be based on the most complex gold standard (score 81 and higher). This research encourages further investigation of gold standards in different cultures.

## Introduction

Workplace bullying is characterized by persistent and systematic negative acts that are directed toward one or more employees by one or more of their colleagues (Einarsen et al., 2009). There are numerous studies that deal with the negative impact of workplace bullying on health and well-being of employees (e.g., Leymann and Gustafsson, 1996; Mikkelsen and Einarsen, 2001, 2002; Matthiesen and Einarsen, 2004; Nielsen et al., 2014; Di Marco et al., 2016; Giorgi et al., 2016b). It can also have negative impact on organizations, leading to a plethora of counterproductive behaviors (Ayoko et al., 2003; Djurkovic et al., 2008; Hoel et al., 2011). Both the personal and organizational outcomes of workplace bullying are of high importance to individual employees, organizations and society at large (Leymann, 1990; Hoel et al., 2001; Giga et al., 2008).

Researchers and practitioners need ways of detecting different categories of employees according to their exposure to negative acts and psychological experience of bullying. Introducing the ROC analysis to identify the victims in different stages of workplace bullying, Notelaers and Einarsen (2013) opened a new chapter in workplace bullying research. In composing gold standard for identifying victims, Notelaers and Einarsen (2013) combined self-labeling with perceived health. In order to enrich this new “chapter” of research, in this research we aimed to explore, develop and test different gold standards of personal and organizational relevance in a specific cultural and economic context of Serbia.

When dealing with workplace bullying, both researchers and practitioners are first confronted with striking individual outcomes. According to Leymann (1990), the first phase of workplace bullying is linked to conflict and stigmatization, while the last phase is seen as a significant disruption of employee’s personal resources, which leads to endangering his/her health. Some studies have confirmed that workplace bullying was related to subsequent health and, mostly, mental health problems (Nielsen et al., 2014). In majority of studies mental health and subjective health were measured by using depression, anxiety and somatic symptoms as indicators. Searching for workplace bullying studies dealing with well-being, we found that most of them operationalized well-being as subjective mental and somatic health. There are only a few studies that included broader subjective well-being indicators, such as life satisfaction (Bowling and Beehr, 2006; Trépanier et al., 2016).

Having in mind a rich body of literature pointing out various negative individual outcomes of workplace bullying, we propose adopting a broader operationalization of well-being. We see life satisfaction as a measure of well-being that incorporates all aspects of subjective mental and somatic health. Moreover, such measure includes more nuances of psychological experiences and emotional states as an expression of the quality of life. Life satisfaction could also be a relevant and sensitive measure of well-being for studying reactions to workplace bullying. It can finely portray all the sequences of bullying, from an isolated critical incident to severe victimization. In that vein, the model of Erdogan et al. (2012) considered life satisfaction as a key indicator of subjective well-being in the work context. After analyzing numerous research studies of life satisfaction, Erdogan et al. (2012) proposed that the level of life satisfaction could be the consequence of the quality of work life. In Bowling and Beehr’s (2006) research it was shown that workplace bullying was positively correlated with depression, anxiety, burnout, frustration, negative emotions at work, and physical symptoms as ‘negative’ psychological indicators of health and well-being. On the other side, it was negatively correlated with positive emotions at work, life satisfaction, self-esteem, job satisfaction and organizational commitment as ‘positive’ psychological indicators of well-being (Trépanier et al., 2016).

Along with individual outcomes, workplace bullying also negatively affects organizational functioning through behaviors that lead to lower productivity, absenteeism and higher turnover rate (Hoel et al., 2011). We consider that the organizationally relevant outcomes are less highlighted in understanding workplace bullying in an individualistic cultural context in which persons are primarily expected to take care of themselves and their close relatives (Hofstede, 2001). As noted by Giorgi (2010), in collectivistic cultures, feeling stronger identification with their organization, employees might perceive negative acts as less harmful than in individualistic cultures and they might be better in coping with negative acts. In support with this thesis, exploring employees’ reactions to severe economic downturn in Serbia it was found that employees were coming to work regularly without receiving salaries for months, as they were strongly attached to their organizations (Čizmić et al., 2004). Both from the theoretical and practical perspective it is important for an organization to explore in depth organizationally relevant outcomes that are crucial for understanding and prevention of bullying.

Embedded in Leymann’s (1990) writings, the atmosphere of exclusion from work could be regarded as ingrained in negative acts such as ignoring and socially isolating the victim that directly signal to an employee that he/she should quit the job. If an organization fails to intervene adequately, the employer loses the employee either by having a detached, dissatisfied, disengaged and an employee of ill-health or having a higher turnover rate since a victimized employee could actually leave the organization (Glambek et al., 2014). Indeed, some authors agree that leaving the organization could be the most effective strategy of coping with severe workplace bullying (Berthelsen et al., 2011).

There is evidence that throughout the entire bullying process employees feel insecure about their jobs and often (re)think themselves to leave the job voluntarily due to unmanageable pressure (Glambek et al., 2014). The results of the cross-sectional research of Glambek et al. (2014) on Norwegian offshore workers showed that exposure to bullying behavior in two different time points was significantly related to job insecurity and employees’ intention to leave the job.

Intention to leave, as any other “intention to…” type of variables, does not indicate actual turnover (Dalton et al., 1999). Based on meta-analysis of 29 research studies carried out in the USA, the mean correlation between intention to leave and turnover rate weighted by the sample size and adjusted for unreliability was -0.32 (Carsten and Spector, 1987). Berthelsen et al. (2011) actually explored turnover as a response to exposure to workplace bullying behaviors. Berthelsen et al. (2011) found that employees that were exposed to bullying behaviors at one point of time reported actually changing the job at a later point in time.

Meta-analyzing correlations between intention to leave and actual leaving the organization, Carsten and Spector (1987) found that this relation was moderated by economic alternatives, i.e., unemployment rate in the society. This is a highly relevant issue for the present study based on research data from Serbia, a country with a very high unemployment rate. Thus, this study puts forward that intention to leave is a persistent outcome of the bullying process and a solid indicator of previous “shock” (Glambek et al., 2014) caused by workplace bullying.

In conclusion, we point out two major outcomes of workplace bullying – intention to leave and well-being. By using these two groups of indicators, in this research we aimed to cover both individual (well-being measured by subjective health and life satisfaction) and organizationally relevant outcomes (intention to leave) of workplace bullying.

### Workplace Bullying in Serbia

Since workplace bullying research has been initiated and well-developed in the Scandinavian cultural context (Einarsen, 2000), there is limited empirical evidence on workplace bullying in other cultural contexts, especially in ‘post-transitional countries’ in Europe (Tambur and Vadi, 2012). Scarce research confirms that bullying may differ from country to country (Jiménez et al., 2007; Lutgen-Sandvik et al., 2007; Baillien and De Witte, 2009; Tambur and Vadi, 2009; Tsuno et al., 2010; Öcel and Aydin, 2012; Seo et al., 2012; Giorgi et al., 2013; Vukelić et al., 2015). Since workplace bullying is a sensitive social phenomenon it is always useful to understand specific national, cultural and economic research context.

Serbia is a country characterized by deep, long-lasting socio-economic crisis that has been progressing since the 1990s as a consequence of economic sanctions and inner political and economic turmoil (Simić et al., 2013). Serbian economy is characterized by low economic activity. According to the 2010 Gallup Wellbeing survey (Gallup, 2010), in terms of life evaluation estimates Serbia was among the top four European countries labeled as the most “struggling” (other two categories being “thriving” and “suffering”). At the time of gathering data for the Negative Acts Questionnaire–Revised (NAQ-R) research presented in this paper, the unemployment rate in Serbia was 22.4% (Statistical Office of the Republic of Serbia, 2012), while GDP per capita was among the bottom 5% of European countries (Eurostat, ND). Apart from being already torn down by inner political and economic situation, Serbia was confronted with the consequences of a deep global financial crisis that begun between 2007 and 2008. Affecting the strongest economies, the financial crisis had a negative impact on labor market across the world (Mucci et al., 2016), and consequently, hampered employees’ well-being (Giorgi et al., 2015a).

In 2010 Serbia adopted the first anti-bullying law that gave the legal and practical reinforcement to the practitioners and researchers of workplace bullying. The law, above all, encourages organizations to work on prevention of workplace bullying. Thus, it is of high importance to constantly follow this phenomenon and identify both organizations and individuals at the risk of being targets of workplace bullying. In the context of direct legal and psychological aid to victims and organizations, it is also important to recognize those severely hurt by workplace bullying.

Workplace bullying has been studied in Serbia since 2009 using The Negative Acts Questionnaire-Revised (Einarsen et al., 2009). Approximately 3,000 employees from different sectors of economy in Serbia took part in several studies of workplace bullying that used the NAQ-R. The overall prevalence of workplace bullying in Serbia, based on the NAQ-R and operationalized as at least two negative acts experienced on a weekly basis is 16% (Petrović et al., 2014). The most frequent negative acts in Serbia are gossiping and rumors, whereas threats of violence and physical abuse or actual abuse are the least frequent. Altogether, person-related bullying is more frequent than the work-related bullying. There were no identified risk groups based on gender, education and hierarchical level. Even though those self-labeled as bullied were significantly older than those self-labeled as non-bullied, the size of the effect was small. Correlating the NAQ-R scores with work related behaviors (Vukelić et al., 2015), the highest correlations were with intention to leave (*r* = 0.457) and perceived organizational support (*r* = -0.497). Correlating the NAQ-R with health and well-being indicators, the highest correlations were with self-rating of health (*r* = -0.315) and satisfaction with life (*r* = -0.273).

### Measuring Workplace Bullying

There are two distinctive ways of measuring workplace bullying (Nielsen et al., 2011): the first one is based on employees’ estimations of the general feeling of being bullied, usually after reading the definition of workplace bullying (the so-called “self-labeling method”), and the second one is based on employees’ ratings of exposure to a range of negative acts that are representative for workplace bullying (the so-called “behavioral experience method”). A significant, but not a complete overlap between the “subjective” and “objective” measures (e.g., Lutgen-Sandvik et al., 2007; Petrović et al., 2014), implies that it is beneficial to apply them both in research of workplace bullying.

Both the Negative Acts Questionnaire (NAQ), and its newest version the Negative Acts Questionnaire–Revised, are probably the most internationally used and most thoroughly psychometrically explored workplace bullying inventories based on behavioral experience (e.g., McCormack et al., 2006; Jiménez et al., 2007; Lutgen-Sandvik et al., 2007; Baillien and De Witte, 2009; Einarsen et al., 2009; Tambur and Vadi, 2009; Tsuno et al., 2010; Öcel and Aydin, 2012; Seo et al., 2012; Giorgi et al., 2013; Notelaers and Einarsen, 2013; Arenas et al., 2015; Vukelić et al., 2015). The NAQ-R was developed based on previous versions of the NAQ scale that went through several quantitative and qualitative research analyses (Einarsen et al., 2009).

Even though the NAQ-R is a very comprehensive measure of workplace bullying with good psychometric properties, there are still plenty of methodological ‘challenges’ (Nielsen et al., 2011). One of the prominent challenges concerns separating victims from non-victims of workplace-bullying. Reviewing published research, we could single out three approaches currently applied in separating workplace bullying victims from non-victims - approach based on the number and frequency of acts, Latent Class Cluster (LCC) approach and Receiver Operating Characteristic (ROC) approach. One approach separates them based on an operational criterion that determines the cut-off point by taking into account the number and frequency of negative acts (Nielsen et al., 2011). However, there is no consensus among researchers about the number and frequency of acts that are critical for separating the victims from non-victims. For example, Leymann (1990) claimed that at least one negative act on a weekly basis could separate the victims from non-victims, Mikkelsen and Einarsen (2001) claimed there should be at least two and Agervold (2007) advocated for at least three negative acts on a weekly bases as a criterion for identifying a victim. Obviously, a relatively arbitrary operational criterion consistent with the reasoning “more negative acts, more often” and treating all bullying behaviors as equally jeopardizing, wasn’t “satisfying” and led researchers to keep on searching for a better solution.

In order to address the limitation of arbitrary cut-off scores, the LCC approach was proposed (Notelaers et al., 2006) as a technique for classifying subgroups of related cases based on experience of different negative acts (Nielsen et al., 2011). A large validation study of the NAQ-R in the UK (Einarsen et al., 2009) found seven emerging clusters based on different levels of exposure to bullying. The clusters varied from “no bullying” to exposure to “severe bullying” and “physical intimidation.” Similar clusters emerged in a study of employees from different Belgian organizations (Notelaers et al., 2006; Einarsen et al., 2009). The Belgian research identified groups from “not bullied,” to groups of those exposed to “limited work criticism,” “work related bulling” and “victims,” with the exception of physical intimidation that has not emerged. In comparison with traditional operational classification method, the LCC demonstrated higher construct and higher predictive validity regarding indicators of stress and well-being (Notelaers et al., 2006). Surpassing the traditional “target–not target” approach, the LCC method offered a variety of target groups based on exposure to negative behaviors (Nielsen et al., 2011). Nevertheless, it did not consider sensitivity or specificity, the basic measures of accuracy in differentiating different clusters (Notelaers and Einarsen, 2013).

### ROC as a Method for Fine-Tuning the Cut-Off Scores

The ROC is often used in medicine for diagnosing the disease (true positive) and correctly rejecting the disease when it is truly absent (Obuchowski, 2003). The ROC is a plot of the sensitivity of a test versus its false-positive rate (1 - Specificity) for all possible cut points. Thus, the area under the ROC curve (AUC) is an indicator of test accuracy or, more precisely, it shows the ability of the test to discriminate between the persons with a certain state or complaint and the persons without it (Hajian-Tilaki, 2013). The impeccable differentiation is reached when the AUC is 1, which means that sensitivity is 1.0, and false positive rate is 0.0 (Obuchowski, 2003). In line with that, the AUC close to 1 indicates high accuracy of the test, while the AUC about 0.5 indicates low accuracy of the test which is almost in line with “chance discrimination” (Hajian-Tilaki, 2013) or close to “chance diagonal” (Obuchowski, 2003).

The ROC analysis is based on an independent criterion of whether or not the individual has some state or disease, the so-called “gold standard” (Streiner and Cairney, 2007). Established on the gold standard, in a process of delineating victims from non-victims, the ROC analysis calculates cut scores on some measure/test under investigation. Logically, it is expected that a gold standard is of an objective nature. However, in the field of organizational psychology, specifically in the area of workplace bullying, it is reasonable to rely upon employees’ subjective perceptions, as an independent criterion (Notelaers and Einarsen, 2013).

Applying the ROC analysis in distinguishing workplace bullying victims from non-victims, Notelaers and Einarsen (2013) operationalized the gold standard based on two indicators—labeling oneself as being subjected to bullying and the personal report of presence of psychiatric symptoms of anxiety and depression. Notelaers and Einarsen (2013) proposed two gold standards: “higher” and “lower.” The lower standard detects employees “in a preliminary stage of bullying” and the higher detects “targets of severe bullying.” More precisely, the lower cut-off point was based on labeling oneself as being bullied at least “now and then” and being a probable “psychiatric case.” On the other hand, the higher cut-off point was based on labeling oneself as being bullied once a week or more often and being a “case in need of treatment.” The precise cut-off NAQ-R points were determined by using the AUC and the highest sum value of sensitivity and specificity as indicators (*Sensitivity* + *Specificity*). They calculated the scores on the NAQ-R in two ways: by adding frequency ratings on all negative acts and by adding previously dichotomized frequency ratings. The frequency ratings were dichotomized as “0,” including the answers “never,” “now and then,” and “monthly,” and “1,” including answers “weekly” and “daily.” Notelaers and Einarsen (2013) determined that the NAQ-R score lower than 33 identifies employees that are not bullied, the score between 33 and 44 identifies those in a preliminary stage of bullying, and the score 45 and higher identifies employees that are victims of workplace bullying. Based on dichotomized frequency ratings they determined the cut score both for lower and higher gold standard, as one negative act that happens once a week or more often.

Departing from Notelaers and Einarsen’s (2013) research, the aim of this study was twofold. First, the broader aim was to propose and test various models of defining the “gold standard” in defining victims in different stages of workplace bullying. In composing gold standards we introduced both personally and organizationally relevant criteria. Apart from combining self-labeling with perceived health status indicator (Notelaers and Einarsen, 2013), we also tested the gold standards constructed based on combining self-labeling with life satisfaction and intention to leave. On one side, we focused on self-rating of health status and satisfaction with life as a broader approach to personal well-being. On the other side, we explored intention to leave as an outcome of workplace bullying that is highly relevant both for the individual employee and for the organization.

The second aim was to answer to Notelaers and Einarsen’s (2013) call for enriching the NAQ-R body of knowledge by determining the cut-off scores for distinguishing the victims of workplace bullying in specific cultural settings. Thus, we wanted to determine the cut-off scores in the Serbian social and cultural context. This research question is interesting as, based on previous research, workplace bullying is more prominent in Serbia than in the Norwegian context analyzed in Notelaers and Einarsen’s (2013) research (Vukelić et al., 2015). In our analysis we followed Notelaers and Einarsen (2013) in determining cut-off scores for both dichotomous and the raw sum NAQ-R scores. We applied a “lower” threshold that could distinguish employees in a preliminary stage of suffering from workplace bullying and a “higher” threshold that could distinguish those that could be regarded as victims of severe bullying.

## Materials and Methods

### Design and Sample

The research is based on re-analyzing the data obtained from a large national workplace bullying survey (Petrović et al., 2014; Vukelić et al., 2015). The data were gathered in cooperation with the Confederation of Autonomous Trade Unions of Serbia that comprises almost one-third of employees in Serbia. Respondents were randomly chosen, regardless of their union membership. Information about participants’ union membership was not collected. The participation in the study was anonymous and voluntary and participants were not rewarded in any way. The study was carried out in accordance with the Code of Ethics (Serbian Psychological Society, 2000).

The sample involved 1,998 employees (54.4% women) from 44 municipalities in Serbia. The mean age of employees was 44.40 (*SD* = 10.23). More than half of respondents completed secondary education (55.5%), almost one quarter had a university diploma (24.3%), 16.2% had trade school/college and 4% had primary education. The majority of employees held subordinate positions (84.7%), while 15.3% were at supervisory positions. More than half of respondents (61.3%) worked in public organizations and 35% were from private organizations. The average length of service with their present organizations was 15.4 years (*SD* = 10.64). Classification of educational attainment applied in this research is somewhat different from the official statistics (Statistical Office of the Republic of Serbia, 2012), but we could conclude that the sample represents population quite well with exception of employees with university education somewhat overrepresented and unskilled employees under represented. Women were slightly overrepresented and age of respondents corresponds with the age structure of the population.

### Instruments and Measures

The NAQ-R (Einarsen et al., 2009) was used to assess the exposure to workplace bullying. The scale consists of 22 items in the form of statements that are rated on a five-point rating scale that denotes the frequency of each negative act within the past 6 months (1 – never; 2 – now and then; 3 – monthly; 4 – weekly, and 5 – daily). The statements cover both direct and indirect negative acts that represent workplace-bullying, without explicitly mentioning the terms “bullying” or “harassment.” The NAQ-R was translated into Serbian using the committee technique in three iterations (Brislin et al., 1973). The psychometric analysis of the NAQ-R in the Serbian population (Vukelić et al., 2015) showed exceptional internal consistency (Cronbach’s alpha of 0.96), as well as satisfactory criterion validity (Vukelić et al., 2015).

The NAQ-R scores were calculated in two ways – as a raw sum of scores on 22 items, and as a sum of dichotomized scores (Notelaers and Einarsen, 2013). The mean of the raw sum scores was 33.67, with a standard deviation of 13.85, and median was at 30.00. Dichotomized scores were determined in line with previous research that counted as bullying at least weekly exposure to negative acts (Leymann, 1996; Notelaers and Einarsen, 2013). The frequency of ratings of negative acts “never,” “now and then,” and “monthly” (ratings 1, 2, and 3) were coded as 0, and “weekly” and “daily” (ratings 4 and 5) were coded as 1. Thus, the mean of sum of the NAQ-R dichotomized scores was 0.99 with a standard deviation of 2.94, and median of 0.00.

We also used the so-called “self-labeling approach,” which is a single item measure of overall victimization from workplace bullying (Einarsen et al., 2009). As a reference, respondents were first presented with the definition of workplace bullying. They rated whether and how much they had been bullied in the past 6 months on the six-point rating scale (No; Yes, very rarely; Yes, now and then; Yes, several times a month; Yes, several times a week, and Yes, almost daily). The mean was 1.49 with a standard deviation of 1.00.

Intention to leave was assessed by the frequency of considering quitting the present job in the past 6 months. It was estimated by one-item on a five-point scale (1 – never, 2 – rarely, 3 – from time to time, 4 – often, 5 – very often). The mean was 1.55 with the standard deviation of 1.03. Health status was also estimated by one item on a five-point scale (1 – very bad, 2 – bad, 3 – neither good nor bad, 4 – good, very good – 5). The mean was 3.62 with standard deviation of 0.84. The ratings for health status were reverse scored for calculating gold standards so that higher score means worse health status.

General satisfaction with life was measured by Diener’s Satisfaction with Life Scale (SWLS, Diener et al., 1985). The scale has five items followed by a seven-point Likert-type scale. Previous research (Vukelić et al., 2015) has shown that the Serbian translation of The Satisfaction with Life Scale had high internal consistency, with Cronbach’s alpha of 0.91. The mean was 17.93 with standard deviation of 7.01. Scores on the SWLS could be interpreted in terms of six categories of life satisfaction: highly satisfied (scores 30–35), high score (scores 25–29), average scores (scores 20–24), slightly below average (15–19), dissatisfied (10–14) and extremely dissatisfied (scores 5–9) (Pavot and Diener, 1993). According to Pavot and Diener’s (1993) classification, the satisfaction with life average score falls in the category “slightly below the average.”

### Operationalizing the “Gold” Standards

Staying in line with the research of Notelaers and Einarsen (2013), for each of the four defined gold standards we performed ROC analyses for higher and lower values of defined variables for both dichotomized and raw scores.

The standards and their lower and higher levels were operationalized as:

(1) Self-labeling and health status

(1) Lower – Self-labeling as being bullied at least “now and then” and estimating health status as at least “neither good nor bad.”
(1) Higher **–** Self-labeling as being bullied at least “several times a week” and estimating health status at least “bad.”

(2) Self-labeling and satisfaction with life

(1) Lower – Self-labeling as being bullied at least “now and then” and having the satisfaction with life scores that could be classified as “slightly below the average” and less satisfied (i.e., categories: slightly below average, dissatisfied and extremely dissatisfied).
(1) Higher **–** Self-labeling as being bullied at least “several times a week” and having the satisfaction with life scores that could be classified as “dissatisfied” and “extremely dissatisfied.”

(3) Self-labeling and intention to leave

(1) Lower – Self-labeling as being bullied at least “now and then” and declaring thinking of intention to leave from time to time and more frequently.
(1) Higher **–** Self-labeling as being bullied at least “several times a week” and declaring intention to leave at least “often.”

(4) Self-labeling, self-perceived health, satisfaction with life and potential intention to leave

(1) Lower – Self-labeling as being bullied at least “now and then,” estimating health status “neither good nor bad” or worse, having the satisfaction with life scores that could be classified as “slightly below average” or lower, and declaring intention to leave from time to time.
(1) Higher **–** Self-labeling as being bullied at least “several times a week,” estimating health status as at least “bad,” having the satisfaction with life scores that could be classified as “dissatisfied” or lower, and declaring intention to leave at least “often.”

In order to perform ROC analyses, first we dichotomized the combination of listed standards that were used as ROC outcome variables so that ‘0’ meant “not at-risk” in respect of the above defined values for each gold standard, and ‘1’ meant “at-risk.”

## Results

In order to test the proposed models of gold standard and determine the cut points that differentiate employees in the preliminary stage of workplace bullying and those that are victims of severe bullying, we performed altogether sixteen ROC analyses. The golden standards and cut-off scores were evaluated and selected by considering two main indicators: AUC (**Table 1**) and the values of sensitivity and specificity and their sum (**Tables 2**–**5**).

**Table 1:** Area under the ROC curve (AUC) scores for tested gold standard models.

	AUC Based on sum of raw NAQ-R scores		AUC Based on sum of dichotomized scores	
		
Gold standard	Lower threshold	Higher threshold	Lower threshold	Higher threshold
Self-labeling and self-perceived health	0.900	0.962	0.799	0.944
Self-labeling and satisfaction with life	0.887	0.957	0.773	0.937
Self-labeling and intention to leave	0.919	0.990	0.859	0.991
Self-labeling, self-perceived health, satisfaction with life and intention to leave	0.917	0.996	0.848	0.995


**Table 2:** Lower threshold (sum of raw scores): cut-off scores for tested gold standard models.

Gold standards	Score	Sensitivity	Specificity	Sens.+Spec.	PPV %	NPV %
Self-labeling and self-perceived health	36	0.902	0.767	1.669	28.17	98.72
Self-labeling and satisfaction with life	34	0.936	0.721	1.657	30.92	98.69
Self-labeling and intention to leave	34	1	0.700	1.694	17.28	100
Self-labeling, self-perceived health, satisfaction with life and intention to leave	40	0.870	0.819	1.689	19.29	99.28


*Sens., sensitivity; Spec., specificity; PPV, positive predictive value; NPV, negative predictive value.*

**Table 3:** Lower threshold (sum of dichotomized scores): cut-off scores for tested gold standard models.

Gold standards	Score	Sensitivity	Specificity	Sens. + Spec.
Self-labeling and self-perceived health	1	0.580	0.894	1.474
Self-labeling and satisfaction with life	1	0.532	0.899	1.431
Self-labeling and intention to leave	1	0.712	0.889	1.601
Self-labeling, self-perceived health, satisfaction with life and intention to leave	1	0.710	0.879	1.589


**Table 4:** Higher threshold (sum of raw scores): cut-off scores for tested gold standard models.

Gold standards	Score	Sensitivity	Specificity	Sens. + Spec.	PPV %	NPV %
Self-labeling and self-perceived health	50	0.882	0.908	1.790	9.15	99.86
Self-labeling and satisfaction with life	67	0.813	0.974	1.787	22.81	99.80
Self-labeling and intention to leave	61	1.000	0.955	1.955	21.28	100
Self-labeling, self-perceived health, satisfaction with life and intention to leave	81	1.000	0.990	1.990	24	100


*Sens., sensitivity; Spec., specificity; PPV, positive predictive value; NPV, negative predictive value.*

**Table 5:** Higher threshold (sum of dichotomized scores): cut-off scores for tested gold standard models.

Gold standards	Score	Sensitivity	Specificity	Sens. + Spec.
Self-labeling and self-perceived health	5	0.882	0.943	1.826
Self-labeling and satisfaction with life	7	0.813	0.962	1.775
Self-labeling and intention to leave	7	1.000	0.966	1.966
Self-labeling, self-perceived health, satisfaction with life and intention to leave	12	1.000	0.985	1.985


As can be seen from **Table 1**, the area under the curve (AUC) values are close to 1, which indicates satisfying differentiation, for both raw and dichotomized NAQ-R scores, as well as for lower and higher threshold approaches. The AUCs were altogether higher for raw sum NAQ-R scores than for dichotomized sum NAQ-R scores indicating the higher accuracy of raw scores, especially for the lower threshold. These findings are in accordance with results from Notelaers and Einarsen’s (2013) study. Also, there was a tendency for the AUCs to be higher for higher thresholds, meaning that higher thresholds produced more accurate classifications.

For the lower threshold, the raw sum score approach produced scores between 34 and 40 (**Table 2**) and the dichotomized score approach produced one-act score (**Table 3**). Based on values of sensitivity, the sum of raw scores approach is more acceptable than the sum of dichotomized scores. On the other hand, specificity values are higher for the sum of dichotomized scores. It means that raw scores identify more accurately true positive cases, while dichotomized scores identify more accurately true negative cases.

Among the tested models of the gold standard, for the lower threshold, the combination of self-labeling (at least now and then) and intention to leave (at least from time to time) produced the highest sum of sensitivity and specificity both for the raw and dichotomized scores approaches (**Tables 2** and **3**). The raw score of 34 produced 0% false negatives and 30.6% false positives for self-labeling and intention to leave as a composite criterion. One negative act on a weekly basis produces 28.8% false negatives and 11.1% false positives for the same criterion.

Concerning the indicators based on the personal perspective that integrate self-perceived health (“neither good nor bad” or worse) or satisfaction with life (“slightly below average” or lower) with self-labeling, the results have shown that the sum of dichotomized scores gives unsatisfying sensitivity. Regarding the sum of raw scores, satisfaction with life combined with self-labeling gives more true positives, while self-labeling combined with self-perceived health gives slightly more true negatives.

For the higher threshold, the raw sum score approach produced scores between 50 and 81 and the dichotomized score approach produced between five and 12 acts score (**Tables 4** and **5**). It is interesting that based on values of sensitivity; both raw and dichotomized scores produced equal proportions of true positives.

As for the specificity, there is no clear difference between the raw sum and dichotomized approaches. Both in case of raw and dichotomized scores approaches, the gold standard that produced the most accurate classifications was the most complex one, a combination of self-labeling (at least “several times a week”), satisfaction with life (“dissatisfied” and “extremely dissatisfied”), self-perceived health (health status as “bad” or worse), and intention to leave (at least “often”). Based on this gold standard, the raw sum score of 81 yielded no false negatives and only 1% false positives. For the same gold standard, the sum of dichotomized scores of 12 negative acts also produced no false negatives and only 1.5% of false positives.

Among the tested gold standards composed of a combination of self-labeling (several times a week or more often) and one more criterion, the combination with intention to leave (i.e., thinking of leaving the organization at least “often”) gave the most accurate classification, as shown by the highest sum of sensitivity and specificity (**Tables 4** and **5**). Comparing the gold standards based on personal perspective that combined self-labeling either with self-perceived health or with satisfaction with life, it is visible that the combination of self-labeling and self-perceived health produced a more accurate classification. For the gold standard defined in terms of self-labeling as being bullied at least “several times a week” and estimating health status as “bad” or worse, the NAQ-R raw sum of 50 and five negative acts on a weekly basis produced less false negatives classifications than the gold standard based on self-labeling and satisfaction with life.

Based on the selected gold standards and selected cut-off scores, we have presented the classification of employees in relation to lower threshold, i.e., being or not being in a preliminary stage of workplace bullying (**Table 6**), as well as in relation to higher threshold, i.e., being or not being a serious victim of workplace bullying (**Table 7**). We can see that based on the lower threshold (**Table 6**), we have almost two thirds of total employees in the true negative category, but almost one-third in the false positive category. It is evident that the cut-off score of 34 for the lower threshold does not miss the employees in a preliminary stage of WPB (**Tables 2** and **6**).

**Table 6:** Classification of employees based on the lower threshold for the self-labeling and intention to leave gold standard.

Gold standard	NAQ-R Raw sum < 33	NAQ-R Raw sum 34 >
Self-labeling as being bullied at least “now and then” (ratings 3–6) and declaring thinking of intention to leave at least from time to time (ratings 3–5)	0.0%	6.6%
Self-labeling as being bullied up to “very rarely” (ratings 1–2, reversed) and declaring thinking of intention to leave rarely or never (ratings 1–2)	61.6%	31.8%


**Table 7:** Classification of employees based on the higher threshold for the composite gold standard.

Gold standard	NAQ-R Raw sum < 80	NAQ-R Raw sum 81 >
Self-labeling as being bullied at least several times a week (ratings 5–6), estimating health status as bad or very bad (ratings 1–2), having the satisfaction with life scores that could be classified as dissatisfied and extremely dissatisfied, and thinking of intention to leave at least often (ratings 4–5)	0.0%	0.4%
Self-labeling as being bullied from no to several times a month (ratings 1–4), estimating health status as neither good nor bad or better (ratings 3–5), having the satisfaction with life scores that could be classified from slightly below average to highly satisfied, and thinking of intention to leave up to from time to time (ratings 1–3)	98.3%	1.3%


Based on the selected gold standard for the higher threshold (**Table 7**), the presented classification is almost impeccable, with only 1.3% of employees that are wrongly classified as victims. Clearly, the low prevalence of workplace bullying needs to be considered and including positive and negative predictive values (NPV) might be useful as they are dependent on the prevalence rate (**Tables 2** and **4**). Positive predictive value (PPV) indicates the likelihood that the employee with the specified NAQ-R raw sum score has been suffering from WPB, and the NPV indicates the likelihood that the employee with the specific score has not been suffering from WPB. It is evident that PPVs for both lower and higher threshold are low (**Tables 2** and **4**). On the other hand, based on NPVs, the selected NAQ-R raw sum scores all give almost 100% likelihood that the employee has not been suffering from workplace bullying, either in the preliminary or severe stage.

## Discussion

Workplace bullying is mostly addressed in the literature for its personal consequences (Hoel et al., 2011). Causing depression, anxiety, burnout, frustration and physical illness, it affects the overall physical and psychological health and well-being (Nielsen et al., 2014). Additionally, it affects organizations in many ways by hampering organizational climate, job satisfaction, work engagement, organizational commitment, organizational and employee well-being, as well as productivity and a number of other aspects of organizational functioning (McCormack et al., 2006; Lutgen-Sandvik et al., 2007; Hoel et al., 2011; Nielsen et al., 2014; Giorgi et al., 2016a; Trépanier et al., 2016). Thus, both from the employee and organizational perspectives, it is vital to detect both the extent of workplace bullying in an organization and the employees that are differently affected by workplace bullying.

From the organizational standpoint, the identification of a category of employees in the preliminary stage of being bullied is very important as the organization could make some changes that could prevent further bulling and subsequent negative feelings. Furthermore, this is an opportunity for the organization to influence the voluntary turnover. The most appropriate gold standard for the lower threshold is the combination of self-labeling and intention to leave (**Table 1**). The NAQ-R score of 34 and higher is the first sign that employees are in the preliminary stage of being bullied. It points to the employees’ overall feeling of being bullied occasionally and considering quitting an organization at least from time to time. Based on the dichotomized scores, the cut value for the lower threshold is the same for all the tested gold standards (one negative act).

For the higher threshold, different gold standards produce more diverse cut scores than for the lower threshold, both for the raw sum and dichotomized sum approaches. The most complex gold standard that entails self-labeling, self-perceived health, satisfaction with life and intention to leave gives the most accurate classifications and the highest cut values for both approaches (**Table 1**). Considering the gold standards that have self-labeling in combination with one more criterion, the NAQ-R sum cut scores are in the range 50–67, while the most complex and composite gold standard gives the highest cut-off value of 81.

Though it is logical to expect that the more criteria the researcher includes, the higher cut value is to be expected, the question is how complex a gold standard should be for the higher threshold. In the composing and subsequent choosing of the gold standard, it is of utmost importance to consider the wider social, economic and cultural context. Keeping in mind that workplace bullying is a complex phenomenon that provokes varied psychological and behavioral responses, it is reasonable to use more criteria to identify victims in a severe stage. Based on small differences in the AUC, sensitivity and specificity values, it could be called into question whether the most complex gold standard gives an adequate return in identifying different categories of employees. We believe that for the higher threshold it is better to apply a more complex gold standard. It gives finer grounds and precision for identifying employees that respond to workplace-bullying in different ways. For the selected most complex gold standard, the higher threshold suggests that the organization should examine all the relevant aspects of the situation at the microscopic level and identify employees that are involved in the situation as well as the particular negative acts.

Considering the classifications of employees based on the lower and higher thresholds, it is evident that the higher threshold for the selected most accurate and at the same time most composite criterion produces the largest proportion of correct classifications with only 1.3% of false positives. The lower threshold also produces 0% of false negative classifications, but it classifies more employees as false positives (31.8%). At the same time, PPVs for the higher threshold indicate that only between 9 and 24% of the time an employee can be accurately classified as being the victim of workplace bullying, whereas there is a 91–76% chance for making a false positive identification. For the lower threshold, PPV indicate that only between 17 and 31% of the time someone identified as being bullied based on a specified NAQ-R score is actually being bullied (the other 83–69% are false positives). Contrary to PPV, the NPVs paint a more precise picture indicating neither of explored thresholds would inappropriately identify someone as being bullied. Taking all together, it should be noted that at the organizational level the lower threshold suggests wider preventive interventions that could not hurt anyone. On the other hand, in-depth exploring the bullying knot and searching for the victims under the microscope could be threatening both for those falsely classified and those affected by false classifications.

Low prevalence rate of workplace bullying makes limited use of identified cut-offs as a predictive tool to identify preliminary and severe workplace bullying victims. Low prevalence rate makes it very hard to positively detect victims of workplace bullying based on any measure (whereas ruling them out is quite precise based on all tested measures). Indeed, low prevalence of workplace bullying was found in a majority of countries where NAQ was used (Zapf et al., 2011; Petrović et al., 2014). However, it should be noted that the aim of using the chosen cut-off scores was not to identify specific victims, but rather to assess the situation in the organization. Thinking in that direction, the proposed cut-off scores can be useful.

Comparing the NAQ-R raw sum and dichotomized sum approaches, our results have indicated that applying raw sum scores in determining cut-off points is superior. It stresses the nature of workplace bullying as not being an either-or phenomenon. The raw sum score approach produces more accurate classifications. As for the lower threshold, the raw sum identifies more accurately the cases that are truly in a preliminary stage of workplace bullying, whereas the dichotomized sum identifies more accurately the cases that are truly not in a preliminary stage of bullying. On the other hand, when it comes to the higher threshold, both raw and dichotomized sums produced equal proportions of true positive classifications. The dichotomized approach may be too rough in identifying employees in a preliminary stage of workplace bullying but more appropriate for identifying severe victims of workplace bullying. Both for the raw and dichotomized sums, the gold standards that rely solely on the personal perspective (self-labeling with self-perceived health and self-labeling with satisfaction with life) gave less accurate classifications, whereas gold standards that included both personally and organizationally relevant perspectives gave more accurate classifications.

Concerning the findings from Serbia and Norway (Notelaers and Einarsen, 2013), the proposed cut-off NAQ-R scores proved to be higher for employees in Serbia. Whereas, based on dichotomized scores, one negative act differentiates Norwegian employees affected with workplace bullying both for the lower and higher thresholds, for the employees in Serbia the cut score for the higher threshold is much higher (from 5 to 12, based on the applied gold standard) than the cut score for the lower threshold. The gold standard tested in this research that is closest to the gold standard applied in Notelaers and Einarsen’s research, i.e., the combination of self-labeling and self-perceived health, still points to five negative acts as a cut-off score. Comparing raw sum data from Serbia and Norway, cut-off scores for the lower thresholds are very close (34 vs. 33), whereas there is a large difference between the cut-off scores for the higher threshold (81 vs. 45). As assessed applying self-labeling and self-perceived health as a gold standard, to affect health it takes more negative acts on a more frequent basis for employees in Serbia than for those in Norway. Of course, comparing the data from Serbia and Norway, we should keep in mind that the applied gold standards were of a different nature, even the closest one that was related to health. What is more important, we should keep in mind that exposure to workplace bullying is more prominent in Serbia than in the Norwegian context (Vukelić et al., 2015). Even though the comparison is not justifiable, it inspires some possible explanations about higher upper threshold scores in Serbia. Since Serbia is highly collectivistic culture, it is possible that colleagues at work could act as a protective social shield from negative acts and thus, more negative acts are needed to provoke more severe consequences. The other possible explanation is tolerance of workplace bulling (Power et al., 2013; Giorgi et al., 2015b). Namely, in some cultures the workplace bullying could be more tolerated than in others. Also, higher upper threshold could be explained by the lower salience of negative acts in comparison with more salient work-related events or events from employees’ personal life. Serbian context is strongly defined by a long lasting economic and social crisis that could easily make existence issues more salient (worrying about satisfying basic needs, etc.) than hardships at work. Deep crisis makes employees more aware and vulnerable to existential stressors putting workplace bullying in the less salient position on the ladder of life stressors.

To sum up, it is once again important to emphasize that this research contributed to Notelaers and Einarsen’s (2013) standpoint of the usefulness of the ROC analysis in defining the cut-off points for the NAQ-R. Unlike other approaches (e.g., LCC, operational criterion), the ROC analysis gives more precise thresholds for separating the victims from non-victims and those in the preliminary stage of being bullied from those that are not. In future developing and investigating of different gold standards it would be useful to find one that could enable a direct comparison of the NAQ-R cut-off points in different cultural settings.

### Strengths and Limitations

In this research, we have answered Notelaers and Einarsen’s (2013) call to explore the ROC in determining the NAQ-R cut-off scores for separating employees in different stages of workplace bullying in a specific socio-economic and cultural context. Presented analyses, notably PPVs and NPVs, clearly indicate that the thresholds found in the current study may not predict victims in different stages of workplace bullying as accurately as one would hope. The presented findings support the need for further research efforts in exploring workplace bullying gold standard.

The findings have also contributed the evidence about the NAQ-R capacity for application in different cultural settings. Additionally, it has been confirmed that it is possible to broaden the NAQ-R application for precisely categorizing employees based on responding to workplace bullying. In this study, we went a step further in testing Notelaers and Einarsen’s (2013) idea by proposing the testing of several gold standard models. In defining the gold standards, we introduced intention to leave as an organizationally relevant criterion since leaving the organization could be one of the most severe consequences of workplace bullying. We have also tried to approach employees’ health from a broader well-being perspective by integrating self-perceived health status and satisfaction with life.

The analysis was performed on a large, well-explored database that was previously used for analyzing psychometric properties of the NAQ-R (Vukelić et al., 2015). The fact that we could not generalize the findings to the Serbian working population, as the sample was not recruited as a representative, could be listed as a potential limitation. However, based on the sampling frame and sample size we expect that it enabled reliable testing of different criteria in determining NAQ-R cut-off scores. Certainly, future longitudinal study could help in unfolding some of the questions posed by this research.

It should be noted that our aim was not to test exactly the same gold standard as Notelaers and Einarsen (2013). For direct comparison purposes, it could be regarded as a limitation of this research. The health status was operationalized as a one-item measure of self-perceived health since some objective measure of employees’ health was not available. Future research could include different indicators of employees’ health.

As for intention to leave as a criterion, it could be observed that different operationalizations are available. In our study, the measure was the frequency of thinking of leaving the organization, whereas in some studies it was the measure of the strength of intention (Carsten and Spector, 1987). In future workplace bullying research, intention to leave could be operationalized both by the frequency of thinking of leaving the organization and by the strength of intention. It is also possible that using different methods, i.e., including objectively measured organizational outcomes (such as actual turnover, employees’ absence from work and productivity loss data) could be useful in objectively specifying the gold standard and further strengthening the organizational perspective in determining NAQ-R cut-off scores. Triangulation could enable determining more robust cut-off scores and also point which of the applied subjective criteria better fitted with the objective indicators of workplace bullying.

Last, but not the least, the nature of explored gold standards can be regarded as a limitation of the study. For the chosen outcome variables (e.g., well-being, intention to leave) we do not have the data that show that these variables are undoubtedly the outcomes of workplace bullying. Even though the indicators of well-being and intention to leave are sensitive to exposure to workplace bullying (e.g., Glambek et al., 2014; Giorgi et al., 2016a), they could also be the indicators of many other processes that happen in organizations. Thus, mix-method and longitudinal studies could help in clearing these questions.

### Implications for Practice and Future Research

Both practitioners and researchers agree on the importance of developing the so-called workplace bullying zero-tolerance organizational climate (Vartia and Tehrani, 2012). Still, before organizations manage to attain this goal, they should successfully deal with workplace bullying on a day-to-day basis. For the purpose of preventing workplace bullying and/or dealing with its consequences it would be beneficial to come up with a cut-off score that would help in detecting not only employees that are under the risk of suffering from workplace bullying (as identified by the lower threshold), but also those that have been severely affected by workplace bullying (as identified by the higher threshold).

Based on our findings we propose organizational interventions at two levels. Departing from the lower threshold, it is advisable to strengthen and introduce more prominent and effective prevention measures. Keeping in mind the severe consequences detected at and above the higher threshold, both from the individual and organizational perspectives, we suggest developing red-alarm, in-depth exploration of the situation and all the involved parties. In published research, the NAQ-R is mostly applied anonymously. The advantages of anonymous organizational surveys are widely accepted. Undoubtedly, in the context of workplace bullying research, anonymity can be regarded as essential both for research participation rate and for employees’ openness and sincerity in answering the survey. However, it is questionable what to do with the survey results if we apply the higher cut-off score and identify severe but anonymous victims of workplace bullying.

The NAQ-R is a widely used workplace bullying instrument with good psychometric characteristics yielded in different cultural settings. It gives solid grounds for comparing countries concerning the rate and different types of workplace bullying acts. Nevertheless, for further cross-cultural exploration it could be useful to check employees’ understanding of rating scale as it is time anchored. As it is known, different cultures have different perception of a time (e.g., Graham, 1981; Wearden, 2016). It is possible that employees from different cultures differently perceive the same frequency of negative acts. To further strengthen golden standards, it would be useful to explore the connections of exposure to negative acts with specific consequences. The researchers should explore whether the health indicators, attitudes and feelings that constitute different golden standards are the consequence of workplace bullying or something else.

At the organizational level NAQ-R can give data about employees’ exposure to specific negative acts and overall intensity of workplace bullying. One of the possible directions for further developing of the NAQ-R could be establishing the proper criterion for identifying victims on a personal level. Yielded results suggest exploring the NAQ-R application as a diagnostic instrument with known participants.

## Conclusion

In this research we wanted to test different gold standards in determining the NAQ-R cut-off scores in Serbia. Workplace bullying as a complex work phenomenon provokes complex experiences and reactions. Exploration of presented different gold standards clearly highlights how difficult it is to identify and detect workplace bullying. The complexity of trying to truly detect and predict possible workplace bullying victims, in the preliminary and especially in the severe stages of bullying, highlights the importance of the current study as well as the importance of continued efforts in this area of research and practice. Seemingly unsatisfactory, the low PPVs call for further efforts in searching for better ways of identifying workplace bullying.

In order to fully grasp the nature of workplace bullying, it is valuable to define and test more gold standards, both from the theoretical and practical standpoints. Our findings confirm that, in addition to Notelaers and Einarsen’s (2013) gold standard based on two criteria – perceived victimization and psychological health, it is reasonable to include more gold standards composed of more criteria and criteria of a different nature. Moreover, the results have confirmed that it was justifiable to compose complex gold standards based on personally and organizationally relevant criteria at the same time. In addition to self-labeling, personal well-being indicators (self-perceived health and satisfaction with life) and intention to leave proved to be important criteria for defining the gold standard.

The cut-off scores determined for employees in Serbia, based on explored gold standards, show that employees in Serbia get into the preliminary stage of feeling bullied based on an almost identical exposure to negative acts at work as employees in Norway, but to feel seriously victimized they need to be much more exposed to negative acts.

The research has presented the application of the ROC analysis in identifying the NAQ-R cut-off scores in Serbia, a specific social, economic and cultural context. The data have confirmed Notelaers and Einarsen’s (2013) proposed approach for exploring the cut-off scores for the NAQ-R, both for the raw sum approach and dichotomized approach, by using the ROC analysis. The results speak in favor of applying the NAQ-R raw sum score approach as superior to the dichotomized sum score approach. As for exploring the lower and higher threshold, our data have confirmed Notelaers and Einarsen’s (2013) reasoning and applications in identifying probable victims in a preliminary stage of workplace bullying as well as severe victims. Presented results strongly point out the difficulties in correctly identifying victims and precision in identifying non-victims of workplace bullying. In closing, this research highlights the need for further exploration of workplace bullying phenomenon in general and gold standard in particular.

## Author Contributions

All three authors, IP, MV, and SČ contributed equally to the research design and writing of this paper. MV conducted the analyses. IP, MV, and SČ are equally accountable for the content of the paper.

## Conflict of Interest Statement

The authors declare that the research was conducted in the absence of any commercial or financial relationships that could be construed as a potential conflict of interest.
